# Modelling the current and future distribution potential areas of *Peperomia abyssinica* Miq., and *Helichrysum citrispinum* Steud. ex A. Rich. in Ethiopia

**DOI:** 10.1186/s12862-023-02177-z

**Published:** 2023-12-06

**Authors:** Debela Daba, Birhanu Kagnew, Belay Tefera, Sileshi Nemomissa

**Affiliations:** 1 College of Natural and Computational Science, Addis Ababa University, Addis Ababa, Ethiopia; 2 Research Department at Gulelle Botanic Garden, Addis Ababa, Ethiopia; 3 College of Natural and Computational Science, Hawassa University, Hawassa, Ethiopia; 4 College of Natural and Computational Science, Kotebe University of Education, Addis Ababa, Ethiopia

**Keywords:** *Ethiopia*, *Habitat suitability*, *Helichrysum citrispinum*, *MaxEnt*, *Modeling*, *Peperomia abyssinica*

## Abstract

**Background:**

The aim of this study is to investigate how climate change influences the distribution of economically and environmentally important species of *P. abyssinica* and *H. citrispinum* in Ethiopia*.* The species distribution modeling intends to forecast species' ecological niche ranges and habitat suitability by employing a variety of environmental parameters as predictors, which is vital for conservation planning and restoration success. Six representative concentration pathways (RCP 2.6, 4.5, and 8.5 for the years 2050 and 2070) with the same resolution of 2.5 min that shows the emission scenarios were used for the prediction. To predict the current and future distributions of *H. citrispinum* and *P. abyssinica* 56 and 45 occurrence records from National Herbarium, Addis Ababa University, GBIF, and available literatures were used respectively.

**Results:**

The MaxEnt model predicted habitat suitability for *H. citrispinum* species with an Area Under Curve (AUC) value of 0.961 ± 0.027, and 0.809 ± 0.045 for *P. abyssinica,* indicating excellent discriminatory ability or accuracy under the current climate scenario. The Future distribution of suitable habitat for both *H. citrispinum* and *P. abyssinica* plant species was accurately predicted with AUC values of 0.960 ± 0.017 and 0.780 ± 0.35, respectively under future climatic scenarios. The jackknife test result indicates that environmental variables such as topographic position index (92.5%), precipitation of the driest quarter (3%) and precipitation in the coldest quarter (1.8%) are associated with the distributions of *H. citrispinum,* while topographic position index (36.6%), precipitation of driest quarter (21.4%), precipitation of warmest quarter (16.2%) and precipitation seasonality (13.9%) were found to be limiting environmental variables for *P. abyssinica* under current and future climatic conditions in Ethiopia. The prediction map and interception calculation for both present and projected (in the 2050s and again in the 2070s) climate change scenarios indicate significant habitat loss, decreased, and fragmentation under all RCPs (2.6, 4.5, and 8.5) scenarios for *P. abyssinica* while habitat gain, and increasing for *H. citrispinum* in Ethiopia.

**Conclusions:**

Topographic position index (TPI) is the most impactful predictor variable on the distribution of the two species. Consequently, potentially habitable areas (with diverse aspects and slopes) are increasing for *H. citrispinum* while decreasing for *P. abyssinica*.

**Supplementary Information:**

The online version contains supplementary material available at 10.1186/s12862-023-02177-z.

## Introduction

Ethiopia is an important regional center of biological diversity, and the flora and fauna have a rich endemic element [1, 2]. The country has the fifth largest flora in tropical Africa. The vegetation types in Ethiopia are highly diverse, ranging from Afro-alpine to desert vegetation. However, the vegetation resources of the country have been reduced due to various factors [2]. According to [3, 4] the most prominent ones are deforestation, expansion of agricultural land, overgrazing, unsustainable utilization, invasion of exotic species, and overexploitation for various purposes such as firewood, charcoal, construction material, and timber, all spurred by rapid human population growth. Modern ecologists, conservationists, and forest managers utilize a variety of modeling techniques, including species distribution modeling (SDM), to show how environmental factors influence past, present, and/or future species distribution patterns. From these species distribution models, BIOCLIM, DOMAIN, GARP, and Maximum Entropy (MaxEnt) are among the algorithms that have been created for modeling species distribution across geographic areas [5, 6]. Even though their performance varies significantly, the majority of these algorithms are user-friendly [7]. The MaxEnt algorithm was selected for this study due to its user-friendliness and popularity as a species distribution modeling algorithm [8]. These techniques have a wide range of uses, particularly in identifying geographic regions where a target species is more likely to be present. Therefore, this study was intended to identify the potential suitable habitats in the face of climate change and dynamic environmental conditions for *P. abyssinica* Miq., and *H. citrispinum* Steud. ex A. Rich in Ethiopia.

The afro-alpine *H. citrispinum* [4] was purposefully selected for modeling potential current and future distribution in the country because of its crucial ecological role as one of the main feed sources [9] for the endemic and endangered *Walia Ibex* [10, 11]. This is aimed at identifying the possible habitats of the species, with the ultimate goal of generating data to ensure proactive conservation measures. This will have a direct positive impact on the conservation and management of *Walia ibex*. These species are dominant in the upper parts of the country and are used to control the ecosystem of the afro-alpine areas. The liquid from the soaked root of *P. abyssinica* is given to pregnant women to avoid malaria [12].

According to [4], *Helichrysum citrispinum* Steud. ex A. Rich belongs to the family Asteraceae and is a cushion-forming perennial herb or shrub that often grows up to 75 cm on rocky mountain slopes and meadows in afro-alpine communities with scattered Erica and Senecios at 3000–4500 m altitudes. It occurs in most highland regions of Ethiopia on open, stony mountain slopes with light loamy soil derived from volcanic sources. In Ethiopia, *H. citrispinum* occurs at Bale, Gondar, Gojam, Shewa around Debre Berhan, and the Arsi Highlands (Chilalo). It is highly distributed on the Afro-alpine belt which is close to the peak of mountain areas like Bale and Ras Dejen. It is also highly distributed in Western African countries like Kenya, Tanzania, and Uganda, but rarely in Namibia and Somalia [4]. However, climate change, anthropogenic factors, and an alarmingly growing population affect the distribution of several plant species, including H*. citrispinum* and *P. abyssinica* in Ethiopia. This plant species distribution is restricted in Ethiopia, and there should be an urgent need for the conservation of these species in Ethiopia.

*Peperomia abyssinica* Miq., which belongs to the family Piperaceae, is a fleshy, succulent perennial herb with trailing or ascending stems rooting at nodes. The stems are usually quite thick and glabrous except for leaf tips [13]. Highly grows in the very driest upland forests, epiphytic or epilithic, usually in the loose litter. It grows at a middle altitude of 1600–3100 m and occurs in most Ethiopian regions. According to the data collected from the National Herbarium (ETH), Addis Ababa, Ethiopia, this species is distributed in the Central, Western, and Eastern parts of the country, especially in the areas classified as Moist Evergreen Africa Forest (MAF) and Dry Evergreen Africa Mountains (DAF). Some examples are Kefa (South Ethiopia), Harenna (Bale), Debra Berhan (Shewa), etc. It is distributed in Western African countries like Zaire, South Sudan, Eritrea, Malawi, and Mozambique [13].

Studying habitat suitability and distribution is the highest and most critical priority for diversity management and developing conservation policies for further action strategies [7, 14]. Therefore, selecting conservation priority sites for plants or animals and developing effective management methods are heavily reliant on accurate data on species distribution and environmental pressures [15]. However, these two species' habitat suitability, distributions, and the current and future impacts of climate change on MaxEnt were studied. From this perspective, it is imperative to conduct research. This study involved model building, validation, and predictions to determine the key climatic factors driving the *Peperomia abyssinica* and *Helichrysum citrispinum (*Supplementary Table S1) distributions and classified the habitats of two species in Ethiopia into ecological types based on climate suitability.

## Methods

### Study area

**Fig. 1:**
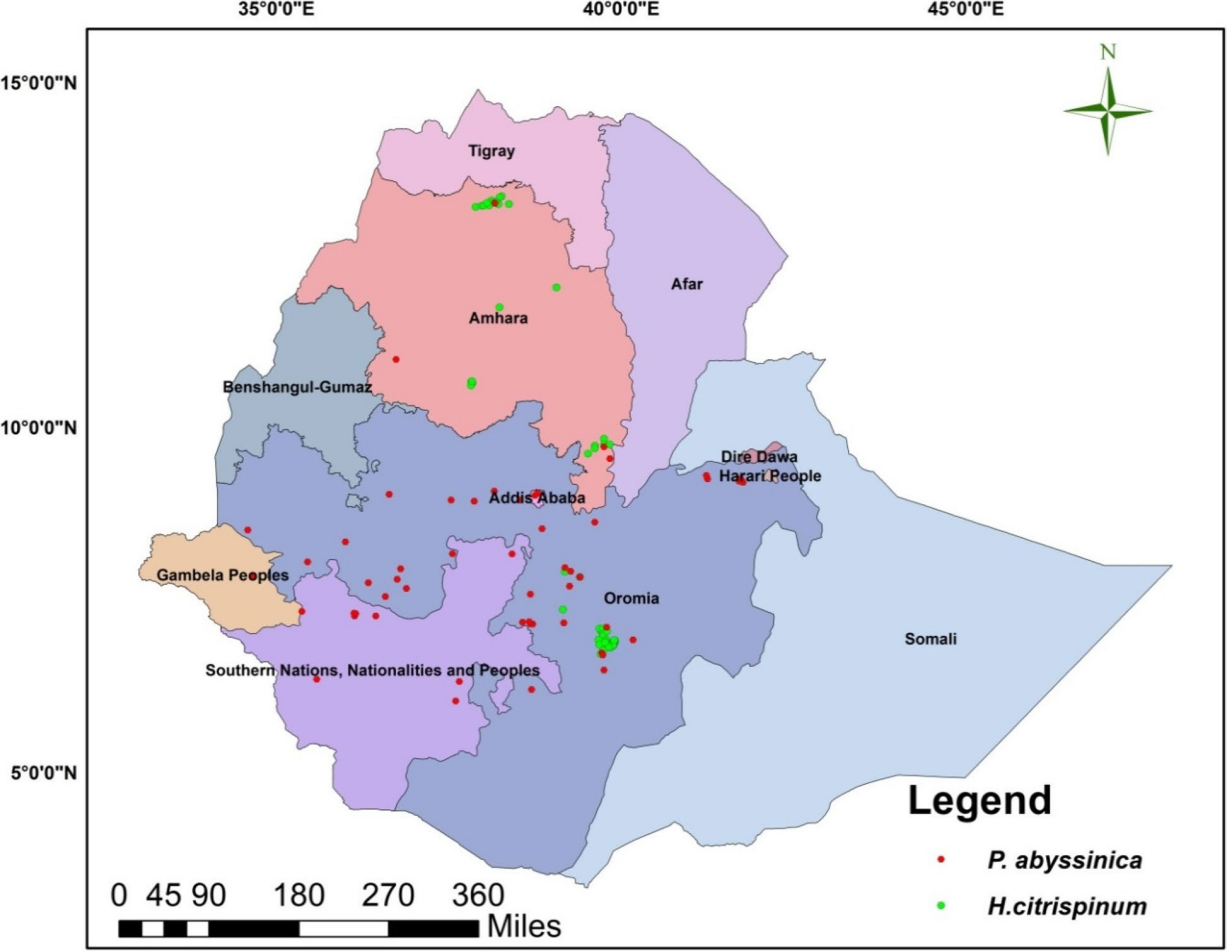
Map of the study area and the sampling points (Source: https://www.mappr.co/political-maps/ethiopia)

The study includes all regions of Ethiopia to project the current and future distribution suitable habitat for both *H. citrispinum* and *P. abyssinica* in Ethiopia (Fig. 1).

### Data analysis

Occurrence data for *P. abyssinica* from field survey (*n* = 20), GBIF (*n* = 480), and the National Herbarium, Addis Ababa University (*n* = 23). The duplicated data’s were removed, and finally, (*n* = 56) (Supplementary Table S2) for *H. citrispinum* and *P. abyssinica* (*n* = 45) (Supplementary Table S3) were used by converting to comma-delimited (CSV) format, and rarified data were used to remove spatially auto correlated occurrence points by reducing multiple occurrence records to a single record within the specified distance. Bioclimatic data with a spatial resolution of 1 km^2^ were obtained from World Climate Version 1.4 for 19 bio variables [16]. In addition to the 19 bioclimatic variables, elevation, Solar radiation [17], and topographic position index (TPI) were included from World Climate Version 2.1. Climatic data are critical for analyzing current and future distribution changes in these two species. The climatic variables were covered from 1960–1990 and included all 19 environmental variables on an annual, monthly, quarterly, and diurnal basis, including minimum, mean, and maximum temperature and rainfall values [16]. A total of 19 climatic variables originally derived from annual, monthly, quarterly, and diurnal temperature and rainfall values collected from weather stations in 2041–2080, were used to depict the future global climate. These layers were extracted and converted to American Standard Code for Information Interchange (ASCII) format using ArcGIS 10.7.1 and used to evaluate the most common environmental variables that contribute biologically to the current and future models of *P. abyssinica* and *H. citrispinum* habitat suitability in the Ethiopian mid and highlands. To identify the relationship between the bioclimatic variable, the multi-collinearity test was performed, and the variables with a coefficient of correlation of *r* < 0.8 were selected (Supplementary Figure S1 a and b) by removing highly correlated variables to avoid over-prediction. For the future prediction, parallel datasets for the global climatic model (CCSM) from three RCPs (2.6-the lowest possible emission scenario; RCPs 4.5-intermediate emission scenario; and RCPs 8.5-the worst case scenario) were used to account for the future distribution of *P. abyssinica* and *H. citrispinum* based on the carbon dioxide emissions for 2050 (average of the predictions for 2041–2060) and 2070 (average of the predictions for 2061–2080) [16].

## MaxENT model building

### Model settings

Before running the MaxEnt model, correlations and the variable inflation factor test were done using R-program version 4.2.0, and environmental variables that were highly correlated (*r* > 0.8) were removed [18, 19]. Such highly correlated variables may result in MaxEnt model overfitting as well as model output interpretation complexity. The MaxEnt algorithm was run with the following settings: 15 replicates, 10,000 background points, 5000 iterations, and a logistic output format. In addition, rarified occurrence data and biased data were used to validate the model’s strength [14].

### Model evaluation

The accuracy of the MaxEnt model was measured by using the area under the receiver operating characteristics curve (AUC), which is a threshold-independent measure of a model’s ability to discriminate between the absence and presence of data [20] and a standard method to evaluate the accuracy of predictive distribution models [21]. An AUC value of 0.5 indicates that the model has no predictive ability, whereas perfect discrimination between suitable and unsuitable cells will achieve the best possible AUC of 1.0. For presence-only occurrence data, AUC can be interpreted as the probability that the model assigns a higher score to a randomly chosen cell known to contain the species than to a randomly chosen cell in which its presence is unknown [22]. 10-percentile training presence logistic thresholds were used to predict habitat suitability and unsuitable distributions of a target species in each scenario, but software defaults were used during the model run [22]. MaxEnt's internal jackknife tests were used to determine the importance of each environmental variable in predicting *P. abyssinica* and *H. citrispinum* distributions in Ethiopia. Out of 22 environmental variables; 11 variables for *P. abyssinica* and 10 variables for *H. citrispinum* were used for MaxEnt algorithm modeling.

### Model prediction

The MaxEnt model has generated one current distribution map and six future distribution maps for each species [23]. Throughout this study, logistic output was chosen because it is simple to understand and can be interpreted as an estimate of the probability of species presence for any given location, ranging from 0 to 1, where 0 represents a very low relative probability of species presence and 1 represents a very high relative probability of species presence [24].

## Result

### Evaluations of the model and variable importance plots of habitat suitability under current climatic condition

**Fig. 2:**
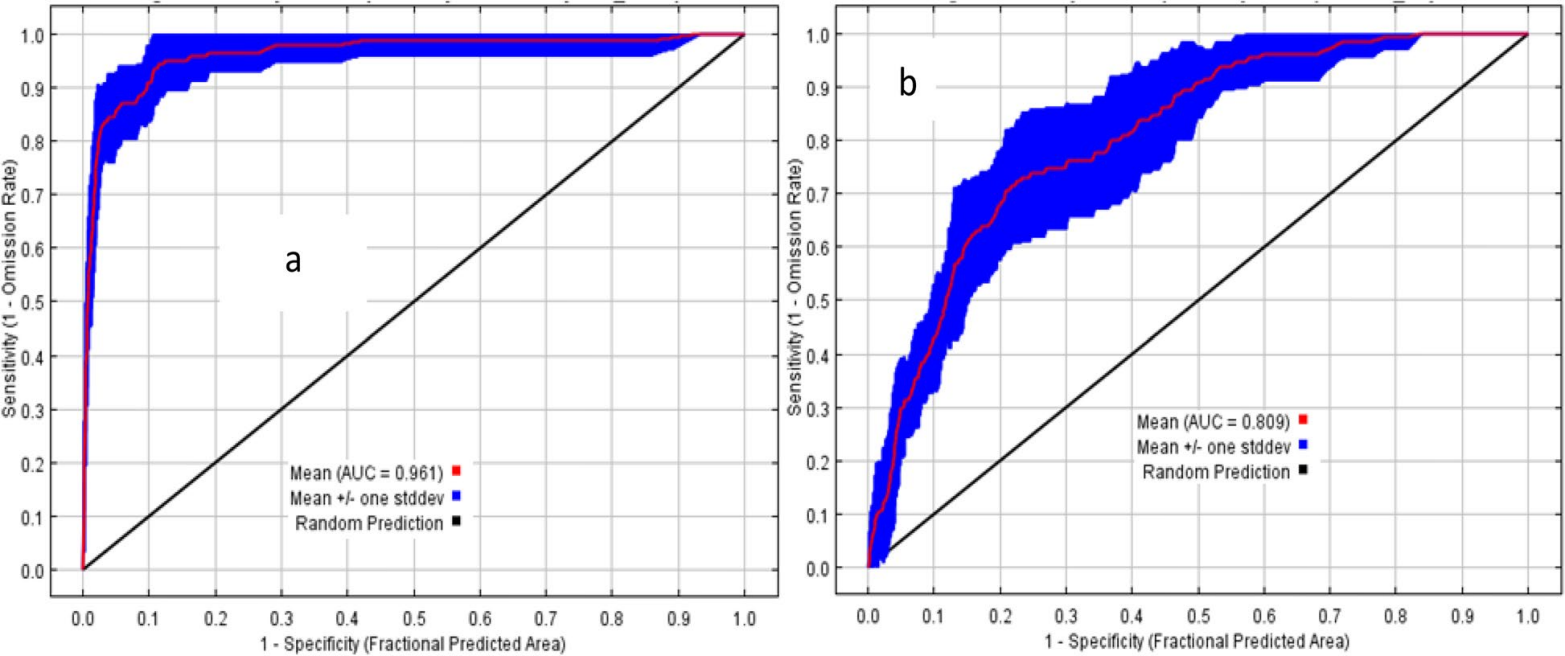
The Receiver Operating Characteristics Curve (ROC) and the value of Area under Curve (AUC) for predicted habitat suitability and distribution of *H*. *citrispinum* (**a**) and *P*. *abyssinica* (**b**)

The AUC value (i.e., the receiver operating characteristic (ROC) curve) for *H. citrispinum* and P. *abyssinica* suitability analyses averaged over 15 replicate runs shows 0.961 and 0.809 (with SD =  ± 1), respectively, revealing an excellent predictive ability to discriminate suitable habitat from unsuitable areas (Fig. 2).

The values displayed are averages of 15 replicated runs with red color indicating the mean of area under curve, blue color showing the mean ± standard deviation, and black color represents random prediction with AUC of 0.5.

The jackknife test variable contributions should be interpreted with caution when the predictor variables are correlated. The values displayed are averages of replicated runs. The findings indicated that *H. citrispinum* current distribution is highly influenced by three variables: the topographic position index (TPI), precipitation in the driest quarter (Bio_17), and precipitation in the coldest quarter (Bio_19), which are explained by 92.5%, 3.0%, and 1.8% contribution (Table 1). The *P. abyssinica* current distribution is highly influenced by four variables: the topographic position index (TPI), precipitation of the driest quarter (Bio_17), precipitation of the warmest quarter (Bio_18), and precipitation seasonality (Bio_15) (Table 2).

**Table 1:** Environmental variable importance for *H*. *citrispinum* distribution in Ethiopia

Variable	Abbreviation	% contribution	Permutation importance
**Topographic Position Index**	**TPI**	**92.5**	**84.6**
Precipitation of Driest Quarter	Bio17	3	2.4
Precipitation in the coldest quarter	Bio19	1.8	2.8
Temperature annual range	Bio7	1	2.8
Precipitation seasonality	Bio15	1	0.5

**Table 2:** Environmental variable importance for *P*. *abyssinica* distribution in Ethiopia

Variable	Abbreviation	% contribution	Permutation importance
**Topographic Position Index**	**TPI**	**36.6**	**21.4**
**Precipitation of Driest Quarter**	**Bio_17**	**21.4**	**0.5**
**Precipitation of Warmest Quarter**	**Bio_18**	**16.2**	**8.3**
**Precipitation seasonality**	**Bio_15**	**13.9**	**31.7**
Solar radiation	Rad	5.3	19.9
Precipitation in the coldest quarter	Bio_19	2	12.7
Temperature Seasonality (standard deviation × 100)	Bio_4	1.7	0.2
Isothermality (BIO2/BIO7) (× 100)	Bio_3	1.6	0.4

**Fig. 3:**
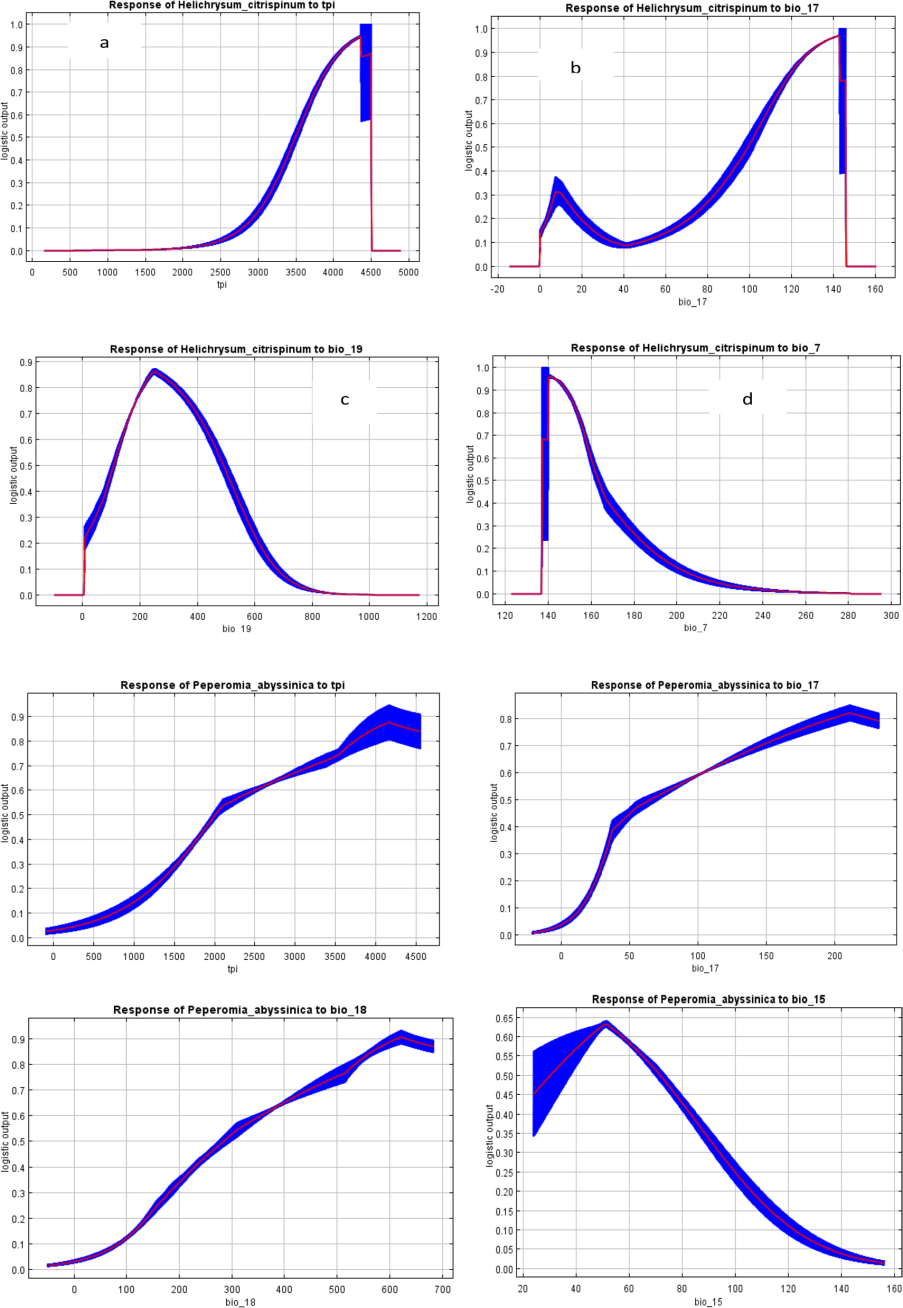
Response curves of the most relevant environmental factors affecting the distribution of *H*. *citrispinum* *(***a**—**d**) and *P*. *abyssinica* (**e**–**h**); the shown values are an average of 15 replicate runs

The response curves indicate how each environmental variable affects the MaxEnt prediction (Fig. 3). The curves show how the predicted probability of presence changes as each environmental variable is varied, keeping all other environmental variables at their average sample value. Note that the curves can be hard to interpret if there is a strongly correlated variable, as the model may depend on the correlations in ways that are not evident in the curves. In other words, the curves show the marginal effect of changing exactly one variable, whereas the model may take advantage of sets of variables changing together. The curves show the mean response of the 15 replicate Maxent runs (red) and the mean ± one standard deviation (blue, two shades for continuous variables).

In comparison to the above marginal response curves, each of the following curves represents a different model, namely, a MaxEnt model created using only the corresponding variable. These plots reflect the dependence of predicted suitability both on the selected variable and on dependencies induced by correlations between the selected variable and other variables. It may be easier to interpret if there are strong correlations between variables.

Based on the species response curves obtained, habitat suitability for *H. citrispinum* (a-d) and *P. abyssinica* (e–h) was positively associated with environmental variables at a certain maximum level, such as topographic position index (Fig. 3a) and precipitation in the driest quarter (Fig. 3b), and negatively associated with precipitation in the coldest quarter (Fig. 3c) and temperature annual range (Fig. 3d), for Helichrysum; Topographic Position Index (Fig. 3e); Precipitation of Driest Quarter (Fig. 3f); Precipitation of Warmest Quarter (Fig. 3g) and positively associated of the environment but Precipitation Seasonality is negatively associated with geographic dstirbution (Fig. 3h) for Peperomia.

**Fig. 4:**
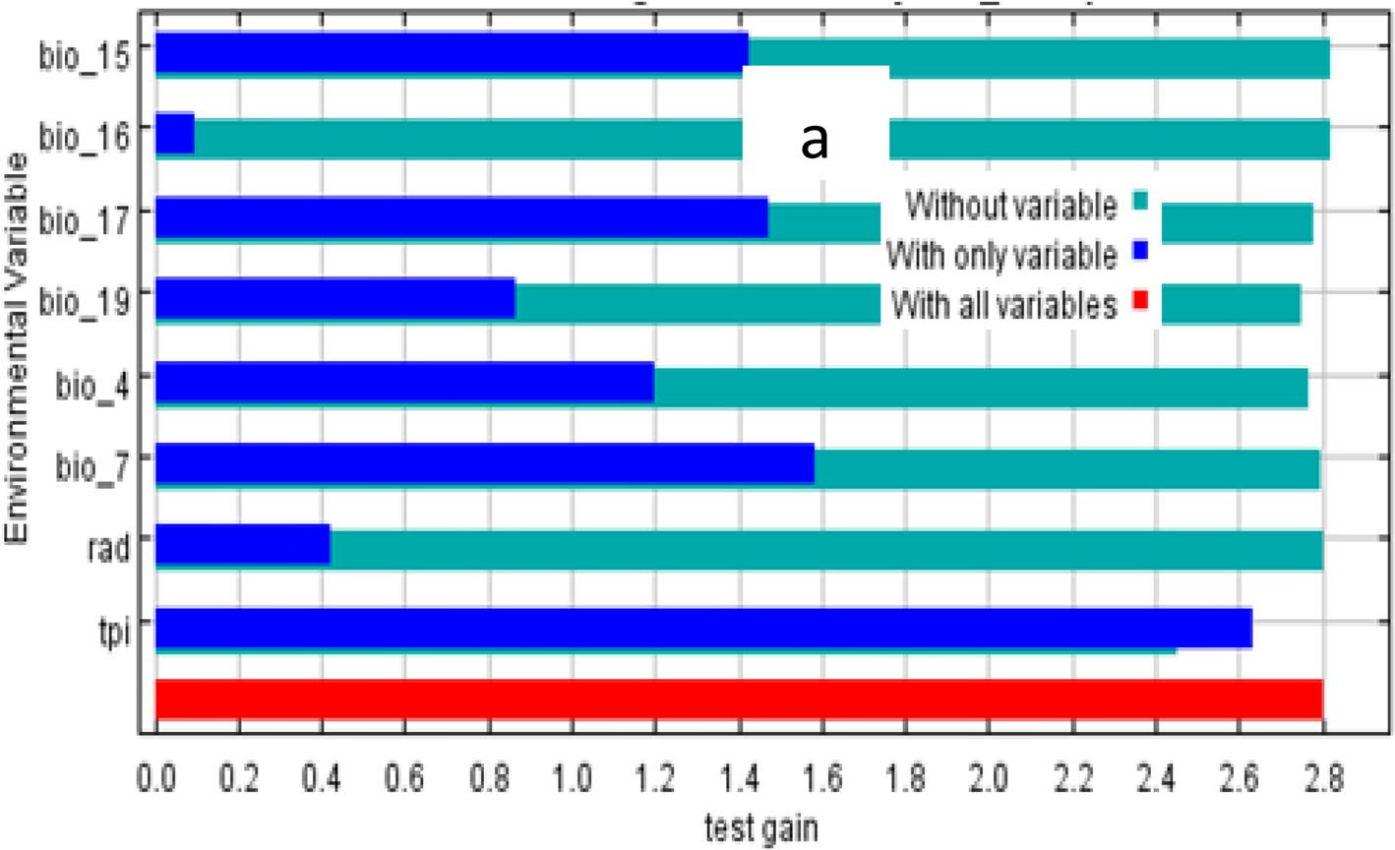
Relative predictive power of different environmental variables based on the jackknife of regularized training gain in MaxEnt models for *H*. *citrispinium*

**Fig. 5:**
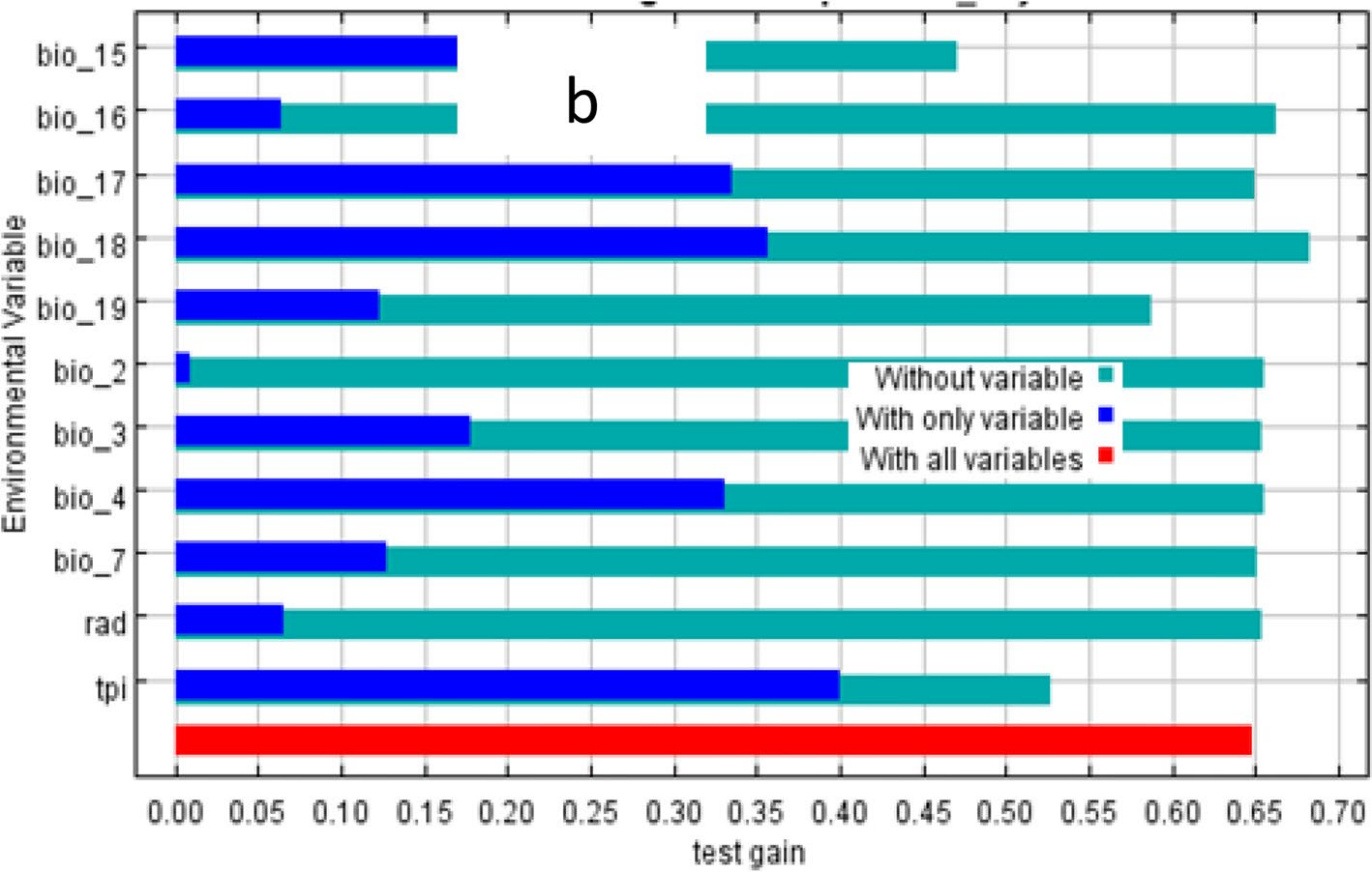
Relative predictive power of different environmental variables based on the jackknife of regularized training gain in MaxEnt models for *P*. *abyssinica*

The test of the jackknife indicates the distribution of both species was mainly influenced by the topographic position index followed by precipitation of driest quarter to the MaxEnt model (Figs. 4 and 5). The values displayed are averages of replicated runs. The blue color indicates the individual variable and the red color indicates the effects of all the predictor’s variables (Figs. 4 and 5).

### Predicted current habitat suitability and distribution of *H. citrispinum* and *P. abyssinica* in Ethiopia

**Fig. 6:**
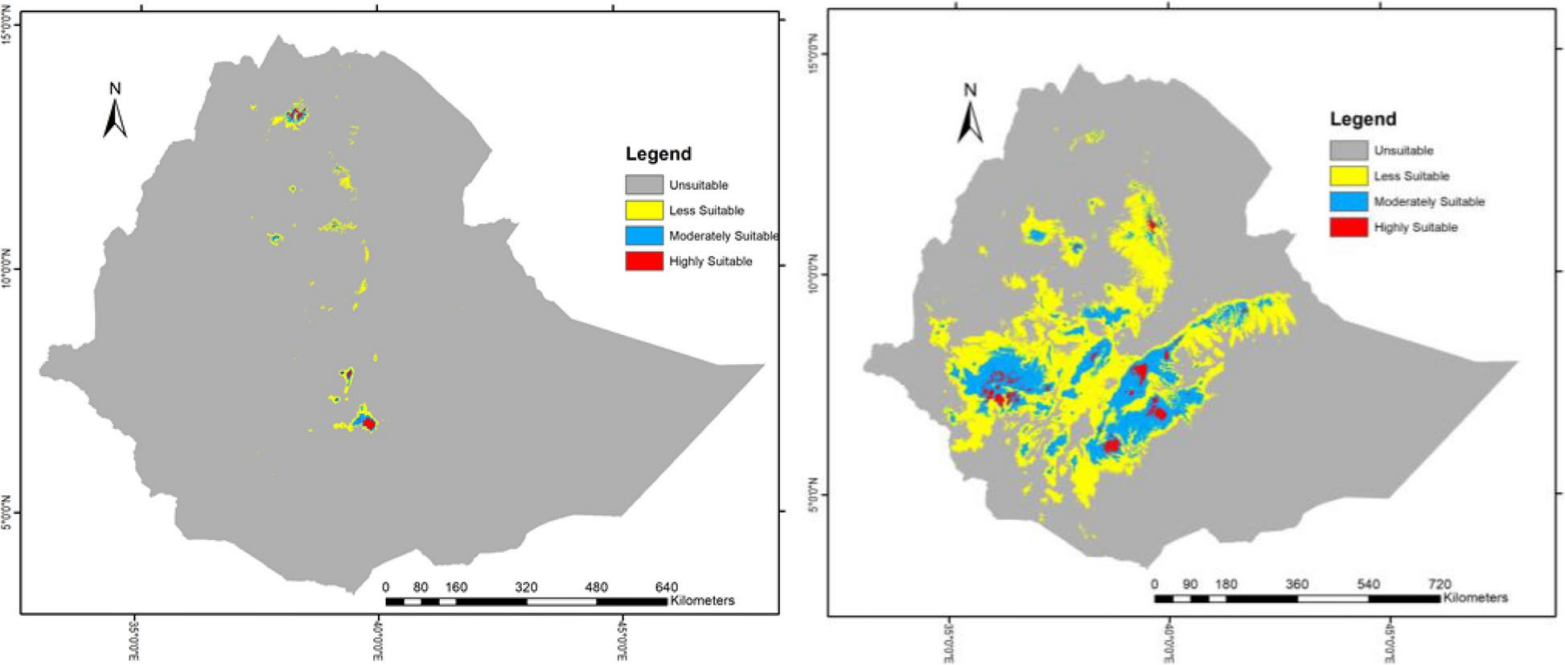
The current potential distribution of the two species in Ethiopia

The habitat suitability and spatial distribution of *H. citrispinum* and *P. abyssinica* were predicted using the predictor variables selected in the process of determining variable importance in the MaxEnt models and uncorrelated predictor variable tests. The accuracy of the distribution maps of *Helichrysum citrispinum* was discriminately very high (AUC = 0.961 ± 0.01) (Fig. 2a) and high for *Peperomia abyssinica* (AUC = 0.809 ± 0.01) (Fig. 2b). Species distribution maps indicated under the current climate scenarios that a total of 972,761.9229 and 24,690,326.65 hectares (100%) of suitable habitat exist for *Helichrysum citrispinum* and *Peperomia abyssinica*, reclassified under the 10% training presence logistic threshold (average threshold = 0.1027 and 0.251), respectively (Fig. 6). The suitable habitat area was further subdivided into highly suitable (0.75–1), moderately suitable (0.5–0.7), and less suitable (0.5) zones for the exact (Supplementary Appendix S1 and S2) areas of two species under different scenarios and years.

### Model evaluations and variables importance to habitat distribution plots of *H. citrispinum* and *P. abyssinica* under future climate scenarios

**Fig. 7:**
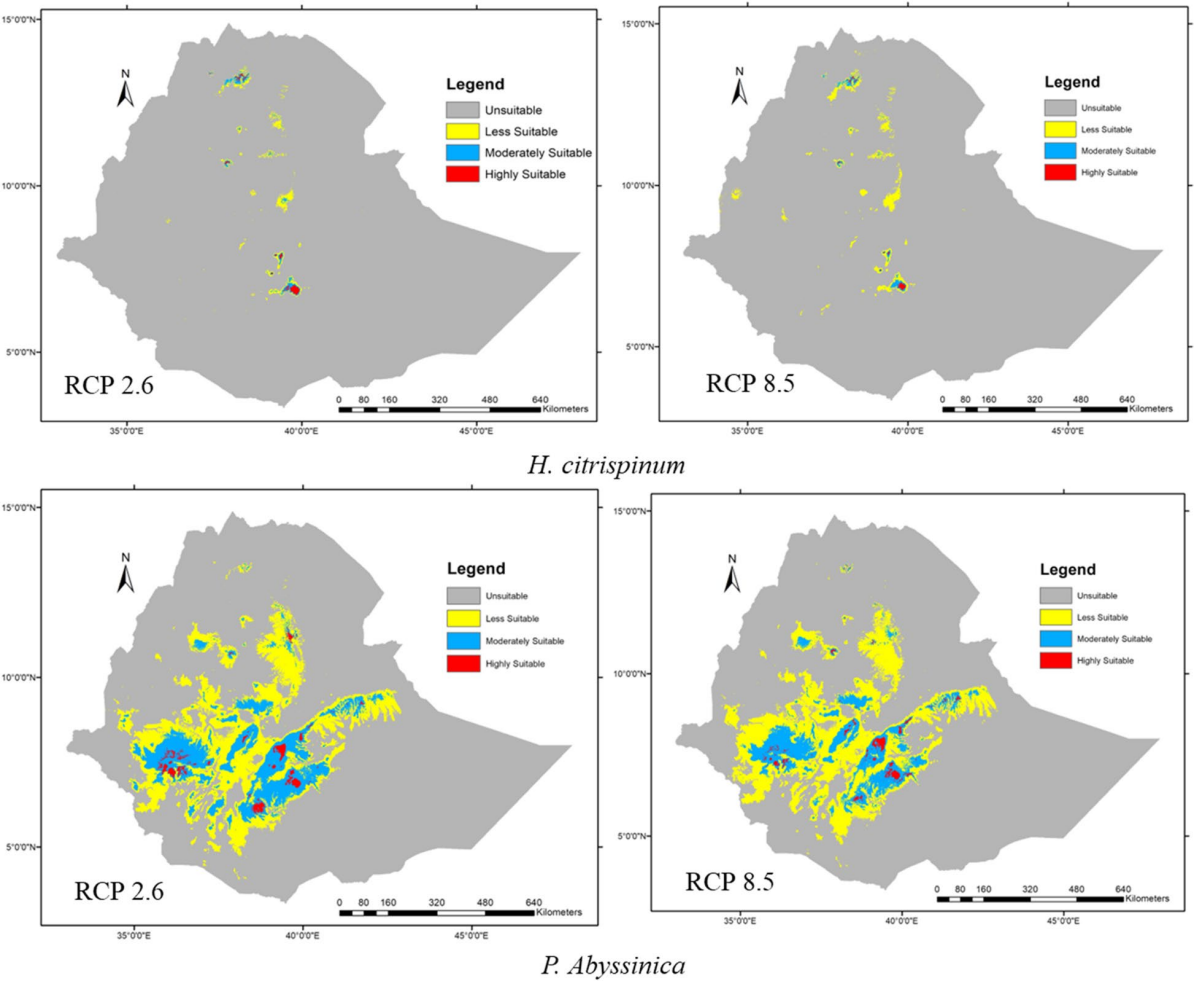
The predicted distribution (2050) of the two species under normal (RCP 2.6) and worst (RCP 8.5) emission scenarios

Future model projections from the 2050s and 2070s obtained from the CCSM climate change scenario indicated that climate change would reduce and shift the target species distribution. However, the impacts are different from one location to another in each scenario. Overall, in response to climate change, the model predicted that highly suitable habitats would be meaningfully changed. The currently suitable area has been significantly reduced, shifted, and fragmented within different scenarios. The effect of climate change on the habitat suitability and distribution of both species under different scenarios, such as current with future 2050s (RCP 2.6) and 2070s (RCP 2.6), current with future 2050s (RCP 4.5) and 2070s (RCP 4.5), and current with future 2050s (RCP 8.5) and 2070s (RCP 8.5), were computed, respectively (Table 3 and Fig. 7).

**Table 3:** the relative percent contributions and importance of bioclimatic variables in Shared Socioeconomic Pathways (SSP) different scenario on *H*. *citrispinum*

**Time**	RCP*	**Variable**	**TPI**	**Bio_3**	**Bio_4**	**Bio_7**	**Bio_15**	**Bio-16**	**Bio_17**	**Bio-18**	**Bio_19**	**Rad**	**AUC ± SD**
Current		%Contribution	92.5		0.2	1	1	0.3	3		1.8	0.3	0.961 ± 0.027
2050 (2041–2060)	RCP2.6	%Contribution	89.8	1.4	0.2	1.1	1.2	0.2	3.7	0.6	1.1	0.6	0.952 ± .029
	RCP8.5	%Contribution	88.6	1.6	0.2	0.5	1.1	0.3	4.2	0.6	1.5	1.2	0.966 ± .021
2070 (2061–2080)	RCP2.6	%Contribution	87.2	1.6	0.1	1.1	1	0.4	4.9	0.7	2.1	0.9	0.960 ± .030
	RCP8.5	%Contribution	85.7	1.6	0.1	1	3.2	0.3	4.7	0.7	1.4	1.2	0.960 ± .020

^*^Representative concentration pathways *AUC the area under the curve *SD Standard deviation

The suitable habitat distribution of *H. citrispinum* and *P. abyssinica* was predicted under the straightest emission pathway scenario (RCP 2.6), and the worst emission scenario (RCP 8.5). The AUC values for different climate change scenarios ranged between current (0.961 ± 0.027) and future (0.952 ± 0.029, which indicate "excellent" predictive power for *H. citrispinum*, and current 0.809 ± 0.045 and future 0.810 ± 0.054 indicate balanced predictive power (Table 3).

The projected distribution of both species under the Community Climate System Model (CCSM, version 4), with RCP 2.6 and RCP 8.5 the habitat suitability is influenced by isothermality (Bio_3) and temperature seasonality (Bio_4) (Tables 3 and 4).

**Table 4:** The relative percent contributions and importance of bioclimatic variables on *P*. *abyssinica*

**Time**	RCP*	**Variable**	**TPI**	**Bio_2**	**Bio_3**	**Bio_4**	**Bio-7**	**Bio-15**	**Bio-16**	**Bio1_7**	**Bio _18**	**Bio_19**	**Rad**	**AUC ± SD**
Current		%Contribution	36.6	0.5	1.6	1.7	0.3	13.9	0.7	21.4	16.2	2	5.3	0.809 ± 0.045
2050 (2041–2060)	RCP2.6	%Contribution	35	0.7	1	3.6	0.2	15.4	0.7	22.7	10.8	3.3	6.5	0.810 ± 0.054
	RCP8.5	%Contribution	34.8	0.3	1	4.5	0	13.7	0.7	19.9	14.5	3.4	6.9	0.799 ± 0.042
2070 (2061–2080)	RCP2.6	%Contribution	34.5	0.5	1.8	3.3	0.1	16.4	0.4	19.4	14.9	3.1	5.7	0.806 ± 0.052
	RCP8.5	%Contribution	38.1	0.5	1.4	1.4	0.2	16.4	1.1	18.8	11.6	3.3	7.2	0.780 ± 0.047

### Predicted future habitat distribution models plots of the species

The predicted change in the habitat distribution of *H. citrispinum* and *P. abyssinica* under the most conventional emission pathway scenario (RCP 2.6) and the worst emission scenario (RCP 8.5) developed by two global climate model systems. The future and current habitat suitability and distribution models were then subtracted from each other, and areas of loss and gain were calculated. The results of the model showed the overall gain and loss in the suitable habitat of *H. citrispinum* and *P. abyssinica* under all future climate scenarios (Fig. 7 and Table 5). The species distribution map revealed that in the mid-century (2050), projections of RCP 2.6 and 8.5 scenarios of the study area were identified as gains or high potential habitats for *H. citrispinum*, which were 18.37% and 73.10%, respectively (Fig. 7 and Table 5), but projections for high potential habitats of *H. citrispinum* in the mid-century (2050) scenarios of RCP 4.5 were reduced or lost by 1.83%, but the species distribution map revealed that in the mid-century (2050), projections of RCP 2.6 and 8.5 scenarios of the study area were identified as reduced for *P. abyssinica* habitat suitability by 8.74%, 5.20%, and 6.43%.

**Table 5:** Predicted change in the habitat distributions of *H*. *citrispinum and P*. *abyssinica* in Ethiopian

Time	RCP*	Predicated habitat suitability *H*. *citrispinum* (million ha)	Predicated habitat suitability *P*. *abyssinica* (million ha)
**Current**		0.97	24.69
**2050s**	RCP2.6	1.15	22.53
	RCP8.5	1.68	23.10
**2070s**	RCP2.6	1.52	22.53
	RCP8.5	1.32	22.81

**Fig. 8:**
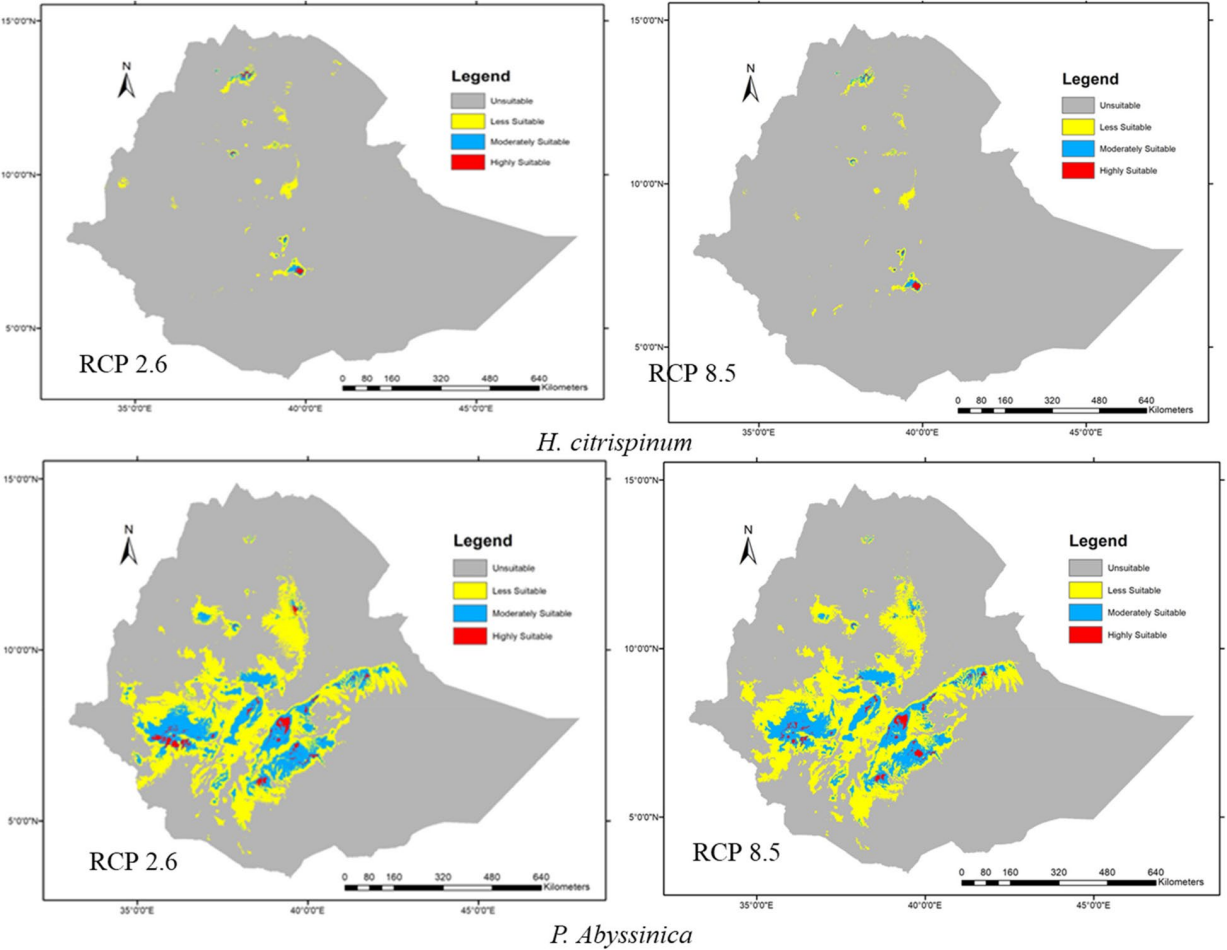
The predicted distribution (2070) of the two species under normal (RCP 2.6) and worst (RCP 8.5) emission scenarios

MaxEnt's future predictions for CCSM emission scenarios of RCP 2.6, 4.5, and 8.5 in the 2070s gain *H. citrispinum* habitat suitability by 55.84%, 51.56%, and 35.36% (Fig. 8), respectively, and projections of the RCP 2.6 and 8.5 scenarios of the study area were identified as loss potential habitats for *P. abyssinica*, which were 8.74% and 7.63%, and the mid-scenario RCP 4.5 gained high potential with 64.46%. MaxEnt’s model output confirms that the habitat suitability of the target species is clearly fragmented, reduced, and completely shifted or extinct in some parts of Ethiopia (Table 5).

Predicted for *H. citrispinum and P. abyssinica* under low (RCP 2.6) and high (RCPP 8.5) emission scenarios by 2050s and 2070s using the global circulation model developed by the Community Climate system Model (CCSM, version 4), which is a climate change model based on the report of the fifth inter-governmental panel on climate change (IPCC).

## Discussion

The habitat suitability and distribution of *H. citrispinum* and *P. abyssinica* in Ethiopia were predicted accurately by the Maxent model. Climatic conditions, species habitat suitability, and occurrences are all important factors in enabling targeted species protection and restoration and guiding landscape strategies [25]. Habitat suitability models improve understanding of the ecology of a targeted species and are effective tools for guiding land-scale choices [26, 27]. Even though several environmental variables govern the habitat suitability of both *H. citrispinum* and *P. abyssinica* in Ethiopia, the main distribution driver for both plant species was found to be TPI, which contributes 92.5% for Helichrysum and 36.6% for Peperomia, followed by Bio_17 (3%, 21.4% for both species, respectively). *H. citrispinum* shows a relatively increasing distribution pattern, attributed to the fact that the species is commonly found in highland areas of the country with diverse landscape features. In addition, *H. citrispinum* is a high-altitude species. Such species residing in mountainous or high-altitude regions may benefit from climate change-induced melting of glaciers and snowpack. As these habitats shrink, species that are adapted to higher elevations may find expanded ranges and increased access to resources.

The MaxEnt model result revealed that the topographic positioning index (TPI) and precipitation of the driest quarter (bio-17) define the potential habitat distribution of these two species, followed by precipitation in the coldest quarter (bio-19) and temperature annual range (bio-7) for *H. citrispinum* and precipitation of the warmest quarter (bio-18) and precipitation seasonality (bio-15) for *P. abyssinica* in Ethiopia. Temperature, precipitation, topography, and soil all influence tree species distribution in South Asia [28]. Habitat suitability is significantly affected by geomorphology, annual precipitation, and ecoregion, which influence plant structure and arrangement [29]. Climate and land-cover changes will have an immediate influence on future species distributions, causing range expansions, reductions, and local extinctions. Nevertheless, evaluations of future range changes often take into consideration the landscape matrix's ability to support species dispersion [30].

This study provides maps of H. citrispinum and *P. abyssinica's* potential range of present and future distribution in Ethiopia under different climate change scenarios. Accordingly, the potential distribution range for H. citrispinum includes the northern, central, and southern eastern parts of the Ethiopian highlands, while *P. abyssinica* mainly found in the central parts of the country under current climate change scenarios. The future model projections for the 2050s and 2070s obtained from the CSSM climate change scenarios indicated that climate change would significantly affect the distribution and habitat suitability of both species, but the impacts were serious in the worst scenarios. The negative effects of climate change led to shifts in suitable habitat ranges for species, while the positive effects significantly increased habitat-suitable areas. Climate change has also had a significant impact on biodiversity and ecosystem change [30]. In response to climate change, the model predicted that highly, moderately, and less suitable habitats would be effectively reduced and fragmented in most areas. Due to the impacts of climate change in Ethiopia, the distribution of both H. citrispinum and P. abyssinica's fragmented and restricted to limited areas. Climate change has quantified impacts on the distribution and suitability of plant diversity, such as *Oxytenanthera abyssinica* [31]. Climate change caused significant fragmentation and reductions in the suitability areas of the species distribution. Therefore, our study confirmed that habitat suitability and distribution have been significantly altered over time. Furthermore, several studies reported that in a simulation of the warming climate in the hilly region of Northern Ethiopia, it was discovered that temperature had a favorable effect on the mountain steppe by accelerating the alpine process during the growing season [32, 33]. Global warming encourages the growth of vegetation. However, plants suffered from a constant rise in temperature [34].

## Conclusion and recommendations

The MaxEnt model discriminates between the current and future distributions of *H. citrispinum* and *P. abyssinica* in Ethiopia. The finding indicates that the topographic position index (92.5%), precipitation of the driest quarter (3%), and precipitation in the coldest quarter (1.8%) are variables that define the distributions of *H. citrispinum*, while the topographic position index (36.6%), precipitation of the driest quarter (21.4%), precipitation of the warmest quarter (16.2%), and precipitation seasonality (13.9%) were found to be limiting environmental variables for *P. abyssinica* under current and future climatic conditions in Ethiopia. The result of the species distribution model map also shows that the distribution of *H. citrispinum* and *P. abyssinica* is fragmented under present and future climatic conditions in Ethiopia. Future studies focusing on the documentation of spatially varying intensities of anthropogenic disturbances and the associated socio-economic activities linked to both species are highly recommended.

### Supplementary Information

**Additional file 1:** **Table S1.** The bioclimatic variables used in the model to predict the preliminary model of the current distribution of *Peperomia* *abyssinica* and *Helichrysum* *citrispinum*. **Table S2.** Occurrence data of *Helichrysum* *citrispinum.* **Table S3.** Occurrence data of *Peperomia* *abyssinica*. **Appendixes S1.** Predicted area of *Herichrysum* *citrispinum* under different scenarios. **Appendixes S2.** Predicted area of *Peperomia* *abyssinica* under different scenarios. **Figure S1.** Correlation’s output between the variables used in the distribution modeling of *Helichrysum* *citrispinum* (a) & *Peperomia* *abyssinica* (b).

## Data Availability

The data can be available in additional files and for detail upon request to the Corresponding Author.
