# Supplementary material for: Modelling the current and future distribution potential areas of Peperomia abyssinica Miq., and Helichrysum citrispinum Steud. ex A. Rich. in Ethiopia
Source: BMC Ecol Evol. 2023 Dec 6;23:71. doi: 10.1186/s12862-023-02177-z (PMC10702103; doi:10.1186/s12862-023-02177-z)
Supplement: Supplementary file 1 — Additional file 1: Table S1. The bioclimatic variables used in the model to predict the preliminary model of the current distribution of Peperomia abyssinica and Helichrysum citrispinum. Table S2. Occurrence data of Helichrysum citrispinum. Table S3. Occurrence data of Peperomia abyssinica. Appendixes S1. Predicted area of Herichrysum citrispinum under different scenarios. Appendixes S2. Predicted area of Peperomia abyssinica under different scenarios. Figure S1. Correlation’s output between the variables used in the distribution modeling of Helichrysum citrispinum (a) & Peperomia abyssinica (b). [file 12862_2023_2177_MOESM1_ESM.docx]

**Tables**

Table S1: The bioclimatic variables used in the model to predict the preliminary model of the current distribution of *Peperomia abyssinica* and *Helichrysum citrispinum*

| **Variable** | **Description** |
| --- | --- |
| BIO1 | Annual Mean Temperature |
| BIO2 | Mean Diurnal Range (Mean of monthly (max temp - min temp) |
| BIO3 | Isothermality (BIO2/BIO7) (×100) |
| BIO4 | Temperature Seasonality (standard deviation ×100) |
| BIO5 | Max Temperature of Warmest Month |
| BIO6 | Min Temperature of Coldest Month |
| BIO7 | Temperature Annual Range (BIO5-BIO6) |
| BIO8 | Mean Temperature of Wettest Quarter |
| BIO9 | Mean Temperature of Driest Quarter |
| BIO10 | Mean Temperature of Warmest Quarter |
| BIO11 | Mean Temperature of Coldest Quarter |
| BIO12 | Annual Precipitation |
| BIO13 | Precipitation of Wettest Month |
| BIO14 | Precipitation of Driest Month |
| BIO15 | Precipitation Seasonality (Coefficient of Variation) |
| BIO16 | Precipitation of Wettest Quarter |
| BIO17 | Precipitation of Driest Quarter |
| BIO18 | Precipitation of Warmest Quarter |
| BIO19 | Precipitation of Coldest Quarter |
| TPI | Topographic position index |
| SRad | Solar radiation |
| Eliv | Elevations |

Table S2: Occurrence data of *Helichrysum citrispinum*

| *Helichrysum citrispinum* | | |
| --- | --- | --- |
| No | Longitude | Latitude |
| 1 | 39.70358 | 7.052294 |
| 2 | 39.857912 | 6.839355 |
| 3 | 39.881944 | 6.835265 |
| 4 | 37.885555 | 13.195487 |
| 5 | 39.910018 | 6.908204 |
| 6 | 39.838817 | 6.806141 |
| 7 | 38.147933 | 13.266108 |
| 8 | 38.2625 | 13.349067 |
| 9 | 37.822 | 10.6575 |
| 10 | 39.903864 | 6.878174 |
| 11 | 38.233333 | 13.333333 |
| 12 | 38.105883 | 13.269717 |
| 13 | 39.89735 | 6.8931 |
| 14 | 38.118817 | 13.287983 |
| 15 | 39.868967 | 6.879267 |
| 16 | 38.20225 | 13.25135 |
| 17 | 37.839167 | 10.638167 |
| 18 | 39.8883 | 6.882183 |
| 19 | 39.88045 | 6.844833 |
| 20 | 38.191944 | 13.263814 |
| 21 | 37.97847 | 13.211568 |
| 22 | 38.133635 | 13.258838 |
| 23 | 38.002394 | 13.213986 |
| 24 | 39.683333 | 7.083333 |
| 25 | 38.083333 | 13.216667 |
| 26 | 39.733333 | 7.083333 |
| 27 | 39.616667 | 9.733333 |
| 28 | 39.4 | 7.833333 |
| 29 | 39.733333 | 7 |
| 30 | 38.216667 | 13.233333 |
| 31 | 39.740995 | 6.977895 |
| 32 | 39.789588 | 7.048879 |
| 33 | 37.8197 | 10.6063 |
| 34 | 38.173285 | 13.251517 |
| 35 | 39.78 | 6.92 |
| 36 | 39.67 | 6.92 |
| 37 | 39.51666667 | 9.61666667 |
| 38 | 39.81397778 | 6.83177222 |
| 39 | 39.15234722 | 7.36000833 |
| 40 | 39.75000028 | 9.83333361 |
| 41 | 39.61457778 | 9.69478611 |
| 42 | 39.75000028 | 9.75000003 |
| 43 | 39.83333361 | 9.75000003 |
| 44 | 39.05776389 | 12.02648333 |
| 45 | 38.36826944 | 13.23618333 |
| 46 | 38.05000028 | 13.25000003 |
| 47 | 39.90000028 | 6.91666669 |
| 48 | 37.83333361 | 10.66666669 |
| 49 | 39.81666694 | 6.81666669 |
| 50 | 38.23333611 | 11.74025 |
| 51 | 39.70000003 | 6.71666669 |
| 52 | 39.76666669 | 6.90000003 |
| 53 | 39.68333336 | 6.85000003 |
| 54 | 39.76666669 | 6.88333336 |
| 55 | 39.73472222 | 6.76666669 |
| 56 | 39.18077222 | 7.90877778 |

Table S3: Occurrence data of *Peperomia abyssinica*

| No | Longitude | Latitude | No | Longitude | Latitude |
| --- | --- | --- | --- | --- | --- |
| 1 | 36.1333 | 7.26667 | 24 | 40.1716 | 6.91923 |
| 2 | 38.158 | 9.07267 | 25 | 39.6161 | 8.62611 |
| 3 | 36.8833 | 7.66667 | 26 | 36.4397 | 7.26611 |
| 4 | 37.6 | 6.03333 | 27 | 39.8336 | 9.54528 |
| 5 | 41.7667 | 9.2 | 28 | 38.5333 | 8.95 |
| 6 | 38.7167 | 7.15 | 29 | 39.1667 | 7.1667 |
| 7 | 36.6333 | 9.03333 | 30 | 39.75 | 9.71667 |
| 8 | 36.15 | 7.3 | 31 | 35.3667 | 7.333 |
| 9 | 36.75 | 7.8 | 32 | 36.0003 | 8.34278 |
| 10 | 38.75 | 9.01667 | 33 | 35.5833 | 6.35 |
| 11 | 38.6833 | 7.15 | 34 | 38.5683 | 7.17472 |
| 12 | 36.8 | 7.95 | 35 | 37.5508 | 8.16889 |
| 13 | 41.7083 | 9.21667 | 36 | 35.45 | 8.05 |
| 14 | 34.5828 | 8.51267 | 37 | 36.5794 | 7.54972 |
| 15 | 39.4 | 7.83333 | 38 | 39.75 | 6.4833 |
| 16 | 38.6667 | 7.18333 | 39 | 38.7835 | 9.04593 |
| 17 | 38.6833 | 7.58333 | 40 | 39.2666 | 7.91667 |
| 18 | 38.1667 | 13.25 | 41 | 36.3333 | 7.75 |
| 19 | 41.7333 | 9.25 | 42 | 38.4167 | 8.16667 |
| 20 | 39.1833 | 7.96667 | 43 | 38.7 | 6.2 |
| 21 | 37.5333 | 8.95 | 44 | 41.25 | 9.25 |
| 22 | 34.6667 | 7.83333 | 45 | 39.7872 | 7.10342 |
| 23 | 37.8667 | 8.93333 |  |  |  |

**Appendixes**

Appendixes S1: Predicted area of *Herichrysum citrispinum* under different scenarios

| year | Senarios | Predicted area (Ha) of *Herichrysum citrispinium* | | | Over all Area |
| --- | --- | --- | --- | --- | --- |
|  |  | Less Suitable | Moderately Suitable | Highly suitable |  |
| current | | 663232.1771 | 214002.7872 | 95526.95864 | 972761.92 |
| 2050 (2041-2060) | 2.6 | 716653.3808 | 309188.6404 | 125591.869 | 1151433.89 |
|  | 4.5 | 648308.8234 | 236256.2421 | 70394.52331 | 954959.59 |
|  | 8.5 | 1344979.408 | 268763.1263 | 70082.0571 | 1683824.59 |
| 2070 (2061-2081) | 2.6 | 1194543.621 | 246246.0881 | 75136.47286 | 1515926.18 |
|  | 4.5 | 1214590.091 | 182066.3328 | 77648.29599 | 1474304.72 |
|  | 8.5 | 894832.342 | 210956.1521 | 210956.1521 | 1316744.65 |

Appendixes S 2: Predicted area of *Peperomia abyssinica* under different scenarios

| year | Senarios | Predicted area (Ha) Area of *Peperomia abyssinica* | | | Over all Area |
| --- | --- | --- | --- | --- | --- |
|  |  | Less Suitable | Moderately Suitable | Highly suitable |  |
| current | | 16925069.69 | 7060524.259 | 704732.7033 | 24690326.65 |
| 2050 (2041-2060) | 2.6 | 15591422.48 | 6341292.046 | 600110.0147 | 22532824.54 |
|  | 4.5 | 16626257.52 | 6198983.706 | 579860.397 | 23405101.62 |
|  | 8.5 | 16370381.23 | 6210462.95 | 522503.3997 | 23103347.58 |
| 2070 (2061-2081) | 2.6 | 15591422.48 | 6341292.046 | 600110.0147 | 22532824.54 |
|  | 4.5 | 21320111.59 | 11751096.92 | 7535757.863 | 40606966.37 |
|  | 8.5 | 16252488.5 | 6081058.549 | 471806.5307 | 22805353.58 |

Figure S1: Correlation’s output between the variables used in the distribution modeling of *Helichrysum citrispinum* (a) & *Peperomia abyssinica* **(b).**
